# Changes in and Remission of Body Weight and Eating Disorder Psychopathology in Adolescents with Anorexia Nervosa During and Four Weeks Post Inpatient Treatment

**DOI:** 10.3390/nu18111786

**Published:** 2026-06-01

**Authors:** Elisabeth M. Neumeier, Linus Imken, Vivien Kaiser, Daniel Le Grange, Verena Haas, Christoph U. Correll

**Affiliations:** 1 Department of Child and Adolescent Psychiatry, Psychosomatic Medicine and Psychotherapy, Charité—Universitaetsmedizin Berlin, Corporate Member of Freie Universitaet Berlin, Humboldt Universitaet zu Berlin, and Berlin Institute of Health, Augustenburger Platz 1, 13353 Berlin, Germany; linus.imken@charite.de (L.I.); vivien.kaiser@charite.de (V.K.); daniel.legrange@ucsf.edu (D.L.G.); verena.haas@charite.de (V.H.); christoph.correll@charite.de (C.U.C.); 2 Department of Psychiatry and Behavioral Sciences, UCSF Weill Institute for Neurosciences, University of California, San Francisco, CA 94143, USA; 3 Department of Psychiatry and Behavioral Neuroscience, The University of Chicago, Chicago, IL 60637, USA; 4 German Center for Mental Health (DZPG), 10117 Berlin, Germany; 5 German Center for Child and Adolescent Health (DZKJ), 13353 Berlin, Germany; 6 Department of Psychiatry, The Zucker Hillside Hospital, Northwell Health, Glen Oaks, NY 11004, USA; 7 Department of Psychiatry and Molecular Medicine, Zucker School of Medicine at Hofstra/Northwell, Hempstead, NY 11549, USA; 8 Einstein Center Population Diversity (ECPD), 10117 Berlin, Germany; 9 Center for Psychiatric Neuroscience, Feinstein Institute for Medical Research, Manhasset, NY 11030, USA

**Keywords:** anorexia nervosa, inpatient treatment, adolescents, eating disorder psychopathology, cognitions, Eating Disorder Examination Questionnaire, remission criteria

## Abstract

Objectives: To assess associations between body weight metrics and eating disorder (ED)-psychopathology in adolescents with anorexia nervosa (AN) at baseline and four weeks post-discharge (4-week follow-up) from inpatient psychiatric multimodal treatment (IMT), calculating full and partial body mass index (BMI) percentile/ED-psychopathology remission rates. Methods: Secondary analysis of a prospective observational cohort study in adolescents (12–18 years) with AN-restricting (AN-R)/-binge–purge (AN-BP), and atypical AN (AAN). Body weight metrics and ED-psychopathology (Eating Disorder Examination Questionnaire, EDE-Q) were assessed at baseline and 4-week follow-up. Remission at 4-week follow-up was calculated by applying German-AN-S3-guidelines-based vs. DSM-5 criteria. Results: In 40 adolescents (mean age = 15.6 ± 1.5 years; females = 90%; BMI *z*-score = −2.59 ± 1.07) receiving IMT (median duration = 118 (IQR = 90–150) days), BMI *z*-score increased (−2.61 to −1.04, *p* < 0.001) and EDE-Q *global score* decreased (3.26 to 1.81, *p* < 0.001) from baseline to 4-week follow-up. Greater weight gain/week during IMT had a positive impact on ED-psychopathology at 4-week follow-up. In multivariable analyses, greater BMI *z*-score improvement was independently predicted by lower baseline BMI *z*-score (*p <* 0.001) and fewer baseline comorbid psychiatric diagnoses (*p* = 0.034) (R^2^_Adjusted_ = 0.545). Greater EDE-Q *global score* improvement was independently predicted by typical vs. atypical AN (*p* = 0.005), higher baseline BMI *z*-score (*p* = 0.012), and higher baseline EDE-Q *restraint* (*p* = 0.048) (R^2^_Adjusted_ = 0.376). By applying stricter S3-guideline-based vs. DSM-5 BMI percentile criteria, full BMI percentile/ED-psychopathology remission was lower (12.5% vs. 50.0%, *p* = 0.001), while non-remission was higher (25.0% vs. 5.0%, *p* = 0.001). Conclusions: The complex relationships between body weight metrics, ED-psychopathology, and treatment outcome trajectories in AN, including treatment response and remission criteria, require further investigation.

## 1. Introduction

Anorexia nervosa (AN) is characterized by intentional weight loss, food restraint, fear of weight gain, and disturbed cognitions and perceptions about eating, weight, and shape [1,2,3], severely impairing physiological and mental health. Eating disorders (EDs) have their peak onset in adolescence (median: 15.5 years) [4]. They are associated with poor outcomes and chronicity in a considerable patient subgroup [5], have a high mortality rate of 5.2/1000 patient-years [5], and can lead to >16 years of potential life lost [6], ranking second among all psychiatric disorders [6]. A systematic meta-review [7] found modest efficacy for interventions for patients with EDs. According to a recent meta-analysis of short- and long-term outcomes in patients with EDs [5], over time only 45% recover, and in 23% EDs take a chronic course.

Treatment for patients with anorexia nervosa (AN) targets body weight restoration and a reduction in ED-psychopathology [8]. Prior studies have shown mixed results, with those in the domain of inpatient psychiatric treatment showing either no impact on ED cognitions and perceptions in adolescents with AN [9], or ED cognition and perception remission not occurring until two years after treatment [10], vs. an increase in body weight being accompanied by decrease in ED-psychopathology at the end of inpatient treatment [11,12]. Few studies have focused on interdependences between changes in body weight and ED-psychopathology during inpatient psychiatric treatment [13,14,15]. Full and partial remission of body weight and ED-psychopathology components, and their relationships to changes in body weight and ED-psychopathology are underexplored. While historically, ED remission criteria were solely body weight-based, outcome reporting considering both body weight and ED-psychopathology has been suggested [8]. Full remission of AN [16] is typically defined by attaining a healthy body weight (e.g., ≥95% expected body weight (EBW)), and low ED-psychopathology (e.g., Eating Disorder Examination Questionnaire *global score* (EDE-Q *global score*) ≤ 1*SD* of community norms). For adolescents with AN, German-AN-S3-guidelines [17,18] recommend targeting a body weight of ≥25th BMI percentile for discharge from inpatient treatment, representing the mean BMI percentile when resumption of menses is expected to occur [19,20]. Definitions of body weight remission range from ≥5th BMI percentile to ≥25th BMI percentile [21,22,23,24,25]. Regarding ED-psychopathology remission, various definitions emerged: United States (US) guidelines [26] define an EDE-Q *global score* as ≤1*SD* (i.e., 68th percentile) of community norms [8,16,27], and the United Kingdom National Institute for Health and Care Excellence (NICE) guidelines [28] state a healthy body weight as a treatment key goal without defining specific ED-psychopathology remission criteria. In a comparative analysis by Le Grange et al. [29] across one single cohort of adolescents with AN receiving family-based treatment, remission varied between 21.7 and 81.7% depending on 11 different available definitions of remission. Couturier et al. [16] found a variability of 3–96% in remission rates in adolescents with AN depending on seven different applied criteria. Bardone-Cone et al. [30] emphasized the longstanding lack of a universal and evidence-based definition of remission for all EDs. Currently, DSM-5 [21] is the only diagnostic manual providing partial and full remission criteria in AN: Partial remission from AN is achieved when *criterion A* (body weight < 5th BMI percentile) has not been met for a sustained period, but either *criterion B* (intense fear of gaining weight or becoming fat, or behavior interfering with weight gain) or *criterion C* (disturbance in self-perceived weight or shape) are still present. Conversely, full remission is defined by no longer meeting criteria A–C [21].

In 2013, atypical AN (AAN) emerged as a DSM-5 diagnosis describing patients with ED-psychopathology and significant weight loss without being underweight [21]. AAN has grown rapidly, comprising approximately one third of patients admitted to pediatric inpatient units/ED treatment programs [31,32,33]. The German AN registry recorded that 13.9% of inpatients with restrictive EDs treated in child and adolescent psychiatry were diagnosed with AAN [34]. A dimensional illness conceptualization of AN and AAN has been proposed [35,36]. Including patients with AAN into studies on EDs while keeping a focus on the potential differences in AAN vs. typical AN is important. Closing the gap of understanding about the associations between, and trajectories of, body weight and ED-psychopathology outcomes and remission in response to different treatment strategies for different AN subtypes might help with developing treatments targeting specific domains responsible for treatment success or lack thereof, improving relapse prevention and quality of life in patients with EDs.

To provide a basis for potential treatment improvements, and for the comparability of European and US studies, the present study aimed to systematically examine changes in body weight metrics and ED-psychopathology between baseline and four weeks post inpatient psychiatric multimodal treatment (4-week follow-up) in adolescents with AN-R, AN-BP, and AAN receiving inpatient psychiatric multimodal treatment (IMT). We had the following a priori hypotheses:

1. IMT will have a greater impact on body weight metric improvement than ED-psychopathology improvement until 4-week follow-up.
2. Body weight metrics and ED-psychopathology will be significantly inversely associated at baseline and 4-week follow-up.
3. In multivariable analyses, baseline body weight metrics, baseline ED-psychopathology, other baseline clinical and treatment characteristics will be associated with changes in body weight metrics and ED-psychopathology from baseline to 4-week follow-up.
4. At 4-week follow-up, partial body weight remission will be more frequent than partial ED-psychopathology remission, and full remission will be least frequent since German-AN-S3-guidelines applied in the treatment setting make IMT discharge dependent on reaching a certain body weight threshold without considering ED-psychopathology.

## 2. Materials and Methods

**Study design**: We conducted secondary analyses on data from a prospective observational cohort study on physical activity in adolescents with AN-R/AN-BP, and AAN performed November 2014–July 2018 at the Department of Child and Adolescent Psychiatry, Charité—Universitätsmedizin Berlin [37,38]. The parent study was approved by the Charité—Universitätsmedizin Berlin institutional ethics committee (identification code: EA2/034/14; approval date: 24 June 2014). Patients entered the parent study on IMT admission, patients aged <18.0 years provided written informed assent [37], caregivers of minors or patients aged ≥18.0 years provided written informed consent. Parent study assessments were conducted in accordance with the Declaration of Helsinki on Ethical Principles for Medical Research Involving Human Subjects.

For the secondary analyses, no additional ethical approval was needed in accordance with the German Professional Code for Physicians, §15, Section 1, as we used previously anonymized data that cannot be attributed to a specific person.

**Participants**: The parent study [37,38] from which this secondary analysis originated included 56 patients, aged 12–18 years, admitted to IMT at one child and one adolescent psychiatry inpatient ward at the Charité—Universitätsmedizin Berlin. Patients met AN-R/AN-BP, or AAN criteria according to international statistical classification of diseases and related health problems, 10th revision, German version (ICD-10) [1,39,40]. ICD-10 [39,40] ED and comorbid psychiatric diagnoses were made by a clinical team experienced in ED diagnosis and treatment as part of routine clinical practice. Inclusion criteria for the present analyses were identical to the parent study but also required complete data on body weight metrics (baseline; end of IMT (EOT); 4-week follow-up) and ED-psychopathology (baseline; 4-week follow-up, due to parent study procedures ED-psychopathology was not assessed at EOT). Exclusion criterion was the presence of conditions affecting physical activity (e.g., hemiplegia), matching the parent study [37,38].

**Inpatient psychiatric multimodal treatment (IMT)**: IMT is standard of care for severely ill patients with AN according to German-AN-S3-guidelines [17], targeting medical stabilization and body weight restoration with additional focus on ED-psychopathology and comorbid psychopathology improvement. IMT includes patient-focused cognitive-behavioral or psychodynamic psychotherapy; dialectic behavioral group therapy for EDs (DBT-E) [41]); caregiver/family sessions; body-oriented therapy; relaxation and weight-adjusted sports therapy; and nutrition counseling. IMT is provided by multidisciplinary teams experienced in ED treatment, including child and adolescent psychiatrists and psychotherapists, nurses, childcare workers, body therapists, social workers, and nutritionists [37,38]. According to German-AN-S3-guidelines, target discharge body weight is the 25th body mass index (BMI) percentile (i.e., mean BMI percentile of expected resumption of menses after secondary amenorrhea [19,42]), with possible clinically driven adaptions based on information about premorbid BMI percentile. Patients’ treatment target is gaining ≥500 g/week, pursued by weekly adjusted daily caloric intake requirements and behavioral reinforcement schedules. If medically indicated, sufficient oral food or liquid intake refusal is compensated with oral nutritional supplements (e.g., Fresubin^®^, Fresenius Kabi, Bad Homburg, Germany) or by enteral tube nutrition. As body weight and ED-psychopathology improve, the supervision of six meals/day by nursing staff is gradually reduced, and patients increasingly regain responsibility for less-supervised food intake. According to German AN registry study data, including data from 14 child and adolescent psychiatry university hospital departments and two major non-university hospitals, the median (interquartile range: IQR) IMT duration in 72 children (aged ≤13 years) treated for AN was 17.0 (IQR = 13.0–21.0) weeks, and 17.0 (IQR = 12.0–21.0) weeks in 217 adolescents (aged 14–18 years) with AN [43].

**Assessments: Clinical and illness characteristics**: Information about patient characteristics was obtained from medical records. Body weight and height were measured in undergarments in the morning fasting (baseline; EOT) or in the afternoon (4-week follow-up) using a digital scale (KERN, MCB, Berlin, Germany) and stadiometer (Seca 2016, Hamburg, Germany) [38]. We calculated BMI (body weight in kilograms divided by height in meters squared) percentile, BMI *z*-score, and percent median BMI (%mBMI) at baseline, EOT, and 4-week follow-up utilizing a German reference data base [44].

**Eating disorder psychopathology**: The EDE-Q (German version) [27] is considered the most commonly used ED-psychopathology assessment, allowing self-rating regarding ED-psychopathology symptom frequency and intensity in adolescents and adults with AN over the prior of 28 days, yielding a *global score* and subscores for *restraint*, *eating concern*, *weight concern*, and *shape concern* as a quantitative assessment of ED-related behaviors, cognitions, and perceptions. EDE-Q validity and reliability among adolescents are considered acceptable [45,46]. In the parent study, by design, the EDE-Q was administered at baseline and 4-week follow-up, but not EOT.

**Change in body weight metrics and ED-psychopathology from baseline to 4-week follow-up**: BMI *z*-score increase indicates weight gain from baseline to 4-week follow-up; reduction in EDE-Q *global score* indicates improvement in EDE-Q *global score*, as a lower score indicates less ED-psychopathology. Change in BMI *z*-score was operationalized as BMI *z*-score at 4-week follow-up minus baseline BMI *z*-score, and change in EDE-Q *global score* was operationalized as baseline EDE-Q *global score* minus EDE-Q *global score* at 4-week follow-up. This methodology was used to present greater improvement (BMI *z*-score increase, EDE-Q *global score* decrease) as more positive values for both domains respectively.

**Remission criteria**: We defined remission according to two different and provisionally defined criteria sets and subsequently compared remission rates:

1. German-AN-S3-guidelines-based remission criteria
  - *Body weight remission*: Body weight ≥ 25th BMI percentile, as German-AN-S3-guidelines recommend a body weight ≥ 25th BMI percentile as the minimal healthy body weight for patients with AN [17].
  - *ED-psychopathology remission*: EDE-Q *global score* ≤ 1*SD* of US community norms (2.44) [27,45,47] as an EDE-Q *global score* ≤ 1*SD* of community norms depicts the common US ED-psychopathology remission criterion [26,45,47]. German-AN-S3 guidelines do not provide ED-psychopathology remission criteria [17].
2. DSM-5 remission criteria
  - *Body weight remission*: Body weight ≥ 5th BMI percentile as DSM-5 states the absence of diagnostic *criterion A* (low body weight < 5th BMI percentile for a significant amount of time) [21] as body weight remission.
  - *ED-psychopathology remission*: EDE-Q *global score* ≤ 1*SD* of US community norms (2.44) [27,45,47]. Although the absence of DSM-5 AN *criterion B* (intense fear of gaining weight or becoming fat, or behavior interfering with weight gain) and criterion C (disturbance in self-perceived weight or shape) constitute full ED-psychopathology remission, we measured the ED-psychopathology of criterion B with the EDE-Q, but not the behavioral aspects of AN. We set the same EDE-Q *global score* threshold as for German-AN-S3-guidelines-based remission criteria.

**Statistical analysis**: Normal distribution was tested using the Shapiro–Wilk test. Using paired *t*-test/Wilcoxon test, body weight metrics and ED-psychopathology change over time was analyzed. (Non-)parametric correlational analyses were conducted to assess cross-sectional associations between body weight metrics and ED-psychopathology at baseline and 4-week follow-up. Univariate regression analyses were used to test the relationship of baseline (sex, admission age, age of AN onset, duration of illness, number of previous inpatient psychiatric treatments for AN, specific AN diagnosis (typical AN-R/AN-BP vs. AAN), comorbid anxiety, depression/dysthymia, personality disorder or obsessive–compulsive disorder, number of psychiatric comorbidities, baseline body weight metrics, and baseline EDE-Q *global score* and subscores) and clinical characteristics (weight gain/week during IMT, change in BMI *z*-score from baseline to 4-week follow-up (BMI *z*-score change, increase indicating improvement), change in EDE-Q scores from baseline to 4-week follow-up (EDE-Q score change, decrease indicating improvement)), with changes in body weight metrics and ED-psychopathology 4-week follow-up as dependent variables. To identify independent correlates of changes in BMI *z*-score and ED-psychopathology from baseline to 4W-PD, multivariable, backward elimination linear regression analyses were conducted. We tested for outliers, collinearity, independent errors, random normally distributed errors, homoscedasticity, linearity, and non-zero variance. We adjusted the final modal effect size (R^2^) for the number of included independent variables to correct for sampling bias [48], expressed as R^2^_Adjusted_. Full and partial body weight metric and ED-psychopathology remission and non-remission rates were compared between the two remission criteria using crosstabulations for small samples (Fisher–Freeman–Halton test). Statistical analyses were conducted for study completers except for patient and illness characteristics and comparison between study completers and dropouts. Regarding missing EDE-Q data, calculations were made with the available data, showing sample sizes for each variable as (*n*). Statistical analyses were performed with IBM SPSS Statistics version 29.0.0.0 (242). Tests were two-sided (α = 0.05) and performed without controlling for multiple testing due to exploratory nature of the correlational analyses. In addition, we conducted a post hoc power analysis [49].

## 3. Results

### 3.1. Participants

**Figure 1:**
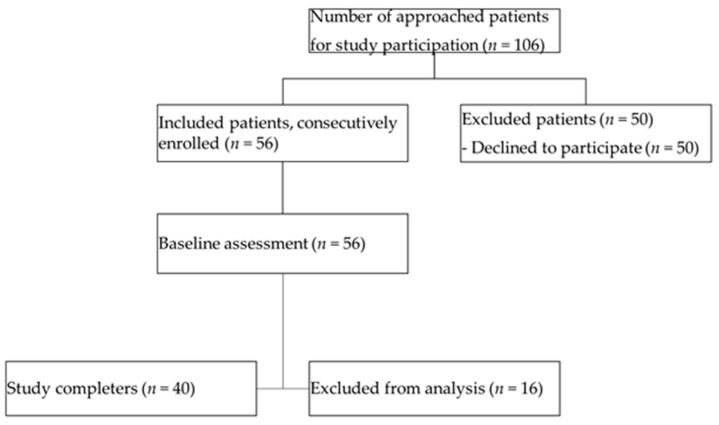
Flow chart of patients included in the study. Abbreviations: *n*, number.

Fifty-six (52.8%) of the initial 106 patients with AN hospitalized for IMT and approached for study participation agreed and were consecutively enrolled (Figure 1). A common reason for declining participation in the parent study was objecting to the recording of physical activity during IMT as a primary study aim. Forty (71.3%) of 56 enrolled patients had the complete baseline, EOT, and 4-week follow-up assessments (study completers) required for the present analyses; 16 dropped out at 4-week follow-up (study dropouts).

### 3.2. Baseline Clinical and Treatment Characteristics

Baseline, clinical, and treatment study sample characteristics are presented in Table 1 and Table A1 (Appendix A). Patients had lost, on average, 12.8 kg between self-reported premorbid and baseline body weight. Six (10.7%) patients were on non-sedating antidepressant medication (sertraline, citalopram) at baseline, and two (3.6%) on antipsychotic medication (i.e., quetiapine, risperidone). Study dropouts (excluded from these analyses due to incomplete body weight metrics and EDE-Q data) had a lower duration of IMT stay and a higher baseline %mBMI. Otherwise, there were no baseline differences between study completers and study dropouts (see Appendix A, Table A1).

### 3.3. Impact of IMT on Body Weight Metrics and ED-Psychopathology

**Figure 2:**
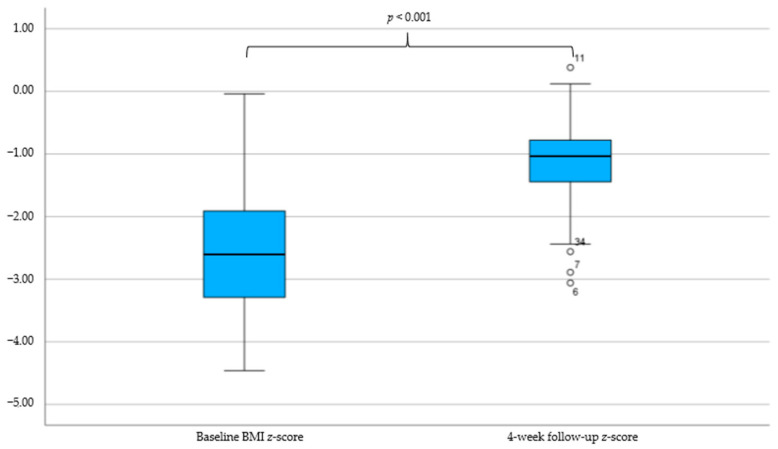
Boxplot of body mass index *z*-score at baseline and four weeks post-discharge from inpatient psychiatric multimodal treatment. Abbreviations: 4-week follow-up, four weeks post-discharge from inpatient psychiatric multimodal treatment; BMI, body mass index.

**Figure 3:**
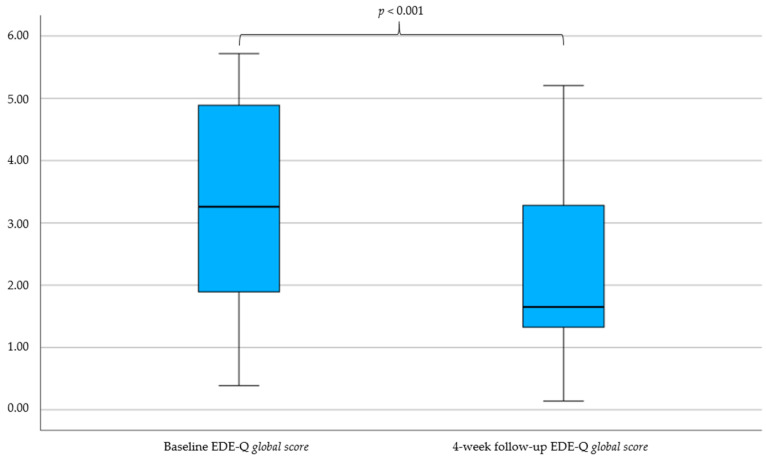
Boxplot of Eating Disorder Examination Questionnaire *global score* at baseline and four weeks post-discharge from inpatient psychiatric multimodal treatment. Abbreviations: 4-week follow-up, four weeks post-discharge from inpatient psychiatric multimodal treatment; EDE-Q, Eating Disorder Examination Questionnaire.

Body weight metrics at baseline, EOT, and 4-week follow-up, ED-psychopathology at baseline and 4-week follow-up, and changes between assessment timepoints are shown in Table 2, Figure 2 and Figure 3.

Fewer patients (5.0%; 2 of 40 adolescents) had a body weight < 1st BMI percentile at 4-week follow-up vs. at baseline (65.0%; 26 of 40 adolescents) (*p* < 0.0001). No patient received enteral nutrition during IMT. Median weight gain/week was 396 (IQR = 280–574) grams/week during a median IMT duration of 117 (IQR = 90–150) days. Body weight metrics increased with large effect sizes between baseline and 4-week follow-up. EDE-Q *global score*, and subscores *weight concern* and *shape concern* decreased with large effect sizes, although effect sizes were numerically smaller than increase in body weight metrics. Effect sizes for decrease in EDE-Q subscores *restraint* and *eating concern* were medium–large.

Higher weekly weight gain during IMT was significantly associated with lower EDE-Q scores at 4-week follow-up: Spearman’s rho showed moderate inverse associations between weight gain in gram/week and EDE-Q *global score* (*p* = 0.039, ρ = −0.328), *weight concern* (*p* = 0.021, ρ = −0.364), and shape concern (*p* = 0.028, ρ = −0.347) 4W-PD. There was no association between baseline ED-psychopathology and weight gain/week during IMT: EDE-Q *global score* (*p* = 0.256, ρ = −0.191), *restraint* (*p* = 0.363, ρ = −0.154), *eating concern* (*p* = 0.280, ρ = −0.188), *weight concern* (*p* = 0.102, ρ = −0.273), and *shape concern* (*p* = 0.220, ρ = −0.207).

### 3.4. Cross-Sectional Associations of Body Weight and ED-Psychopathology at Baseline and 4-Week Follow-Up

There were no cross-sectional associations found between body weight metrics and ED-psychopathology, neither at baseline nor at 4-week follow-up, as shown in Table 3.

### 3.5. Associations of Baseline and Clinical Characteristics with Change in Body Weight Metrics from Baseline to 4-Week Follow-Up

In univariate linear regression analyses, greater BMI *z*-score increase from baseline to 4-week follow-up was significantly associated, among baseline and clinical characteristics (Table 4), with baseline BMI *z*-score (*p* < 0.001), baseline comorbid psychiatric diagnosis of depression or dysthymia (*p* = 0.025), baseline number of baseline psychiatric comorbidities (*p* = 0.036), and typical (AN-R, AN-BP) vs. AAN diagnosis (*p* = 0.049).

A multivariable backward elimination linear regression analysis was conducted to assess if significant variables from univariate regression analyses were independently associated with change in BMI *z*-score, which on average increased by 1.4138 ± 0.88522 during the observation period.

**Figure 4:**
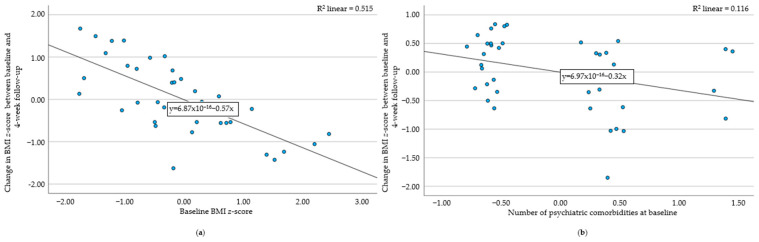
Partial regression plot, dependent variable: change in BMI z-score between baseline and 4-week follow-up, (**a**) independent variable: baseline BMI *z*-score and (**b**) independent variable: number of psychiatric comorbidities at baseline. Abbreviation: 4-week follow-up, four weeks post-discharge from inpatient psychiatric multimodal treatment.

Greater BMI *z*-score increase was independently predicted by lower baseline BMI *z*-score (*p* < 0.001), and fewer baseline psychiatric comorbidities (*p* = 0.034) (model: *n* = 40, R^2^ = 0.568; R^2^_Adjusted_ = 0.545; *p* < 0.001) (Table 5, Figure 4a,b).

Per each point lower baseline BMI *z*-score, the BMI *z*-score from baseline to 4-week follow-up was increased by 0.566 (95% CI, −0.422 to 0.699). Per each one less psychiatric comorbidity, BMI *z*-score from admission to 4-week follow-up increased by 0.317 (95% CI, −0.608 to −0.026).

### 3.6. Associations of Baseline and Clinical Characteristics with Change in ED-Psychopathology from Baseline to 4-Week Follow-Up

In univariate linear regression analyses, greater EDE-Q *global score* reduction (i.e., improvement) from baseline to 4-week follow-up was significantly associated among baseline and clinical characteristics (Table 4) with a higher baseline EDE-Q *global score* (*p* < 0.001), and subscores EDE-Q *restraint* (*p* = 0.003), EDE-Q *eating concern* (*p* < 0.001), EDE-Q *weight concern* (*p* = 0.004), and EDE-Q *shape concern* (*p* = 0.002), typical (AN-R, AN-BP) vs. AAN diagnosis (*p* = 0.018), baseline comorbid anxiety disorder (*p* = 0.068), baseline number of psychiatric comorbidities (*p* = 0.092), and higher baseline BMI *z*-score (*p* = 0.094).

A backward elimination linear regression analysis was conducted to assess if significant variables from univariate regression analyses were independently associated with change in EDE-Q *global score*, which on average decreased significantly by 0.9945 ± 1.28057 during the observation period.

**Figure 5:**
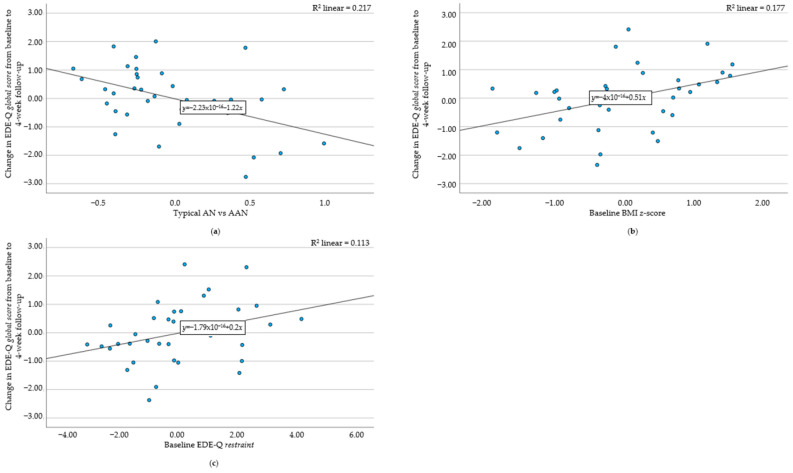
Partial regression plot, dependent variable: change in EDE-Q *global score* between baseline and 4-week follow-up, (**a**) independent variable: typical AN vs. AAN, (**b**) independent variable: number of psychiatric comorbidities at baseline and (**c**) independent variable: baseline EDE-Q *restraint*. Abbreviations: 4-week follow-up, four weeks post-discharge from inpatient psychiatric multimodal treatment; AN, anorexia nervosa; AAN, atypical anorexia nervosa; EDE-Q, Eating Disorder Examination Questionnaire.

Greater decrease in EDE-Q *global score*, equaling more improvement in ED-psychology between baseline and 4W-PD, was independently predicted by ED diagnosis (typical vs. atypical AN) (*p* = 0.005), higher baseline BMI *z*-score (*p* = 0.012), and higher baseline EDE-Q *restraint* (*p* = 0.048) (model: *n* = 37, R^2^ = 0.428, R^2^_Adjusted_ = 0.376, *p* < 0.001) (Table 6 and Figure 5a–c).

By typical AN vs. AAN diagnosis, EDE-Q *global score* change was increased by 1.224 points (β = 1.224, 95% CI, 0.401 to 2.046), i.e., patients with typical AN compared to patients with AAN had a greater reduction in EDE-Q *global score* equaling more improvement in ED-psychopathology from baseline to 4-week follow-up.

Per each one-point-higher baseline BMI *z*-score, the EDE-Q *global score* from admission to 4-week follow-up decreased by 0.508 (β =−0.508, 95% CI, −0.896 to −0.120) equaling more improvement in ED-psychopathology from baseline to 4-week follow-up.

Per each one point higher baseline EDE-Q *restraint* subscale score, EDE-Q *global score* decreased by 0.199 (β = −0.199, 95% CI, −0.397 to −0.002) equaling more improvement in ED-psychopathology from baseline to 4W P-D.

### 3.7. Remission Rates

**Figure 6:**
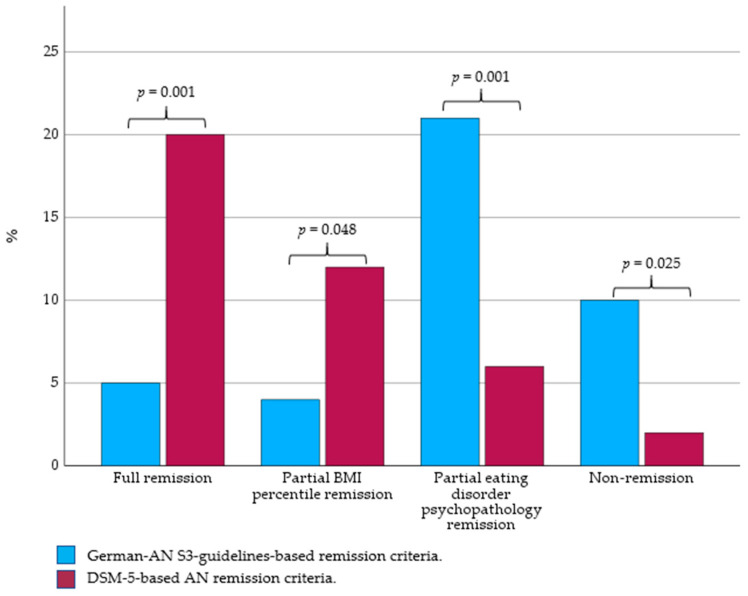
Remission rates four weeks post-discharge from inpatient psychiatric multimodal treatment using German S3-guideline-based vs. DSM-5-based anorexia nervosa remission criteria. Abbreviations: %, percent; AN, anorexia nervosa; BMI percentile, body mass index percentile; *p*, probability value.

Figure 6 shows remission rates at 4-week follow-up applying German-AN-S3-guidelines-based vs. DSM-5-based remission criteria.

When applying German-AN-S3-guidelines-based remission criteria at 4-week follow-up, among 40 patients, 12.5% met full (BMI percentile and ED-psychopathology) remission criteria. Altogether, 10.0% patients were in partial BMI percentile remission, 52.5% in partial ED-psychopathology remission, and 25.0% were in both BMI percentile and ED-psychopathology non-remission.

When applying DSM-5-based remission criteria, 50.0% patients met full remission criteria, 30% were in partial BMI percentile remission, 15.0% in partial ED-psychopathology remission, and 5.0% were in both BMI percentile and ED-psychopathology non-remission.

When comparing the two remission criteria systems, remission rates at 4-week follow-up were higher for full remission (*p* = 0.001) and for partial BMI percentile remission (*p* = 0.048) applying DSM-5-based vs. German-AN-S3-guideline-based remission criteria. Conversely, partial ED-psychopathology (*p* = 0.001) and full non-remission (*p* = 0.025) rates were higher applying German-AN-S3-guideline-based vs. DSM-5-based remission criteria.

### 3.8. Post Hoc Power Analysis

The study was small which limits statistical power. The post hoc power analysis yielded that with α = 0.05 and β = 0.80, the sample size of 40 patients resulted in the ability to detect a minimum correlation coefficient of 0.43.

## 4. Discussion

The main aims of this study were to assess the impact of IMT on body weight metrics and ED-psychopathology, the associations of body weight metrics and ED-psychopathology between baseline and 4-week follow-up, and the associations of baseline and treatment variables with changes in body weight metrics and ED-psychopathology in adolescents with AN-R/AN-BP and AAN receiving IMT. We also explored full and partial BMI percentile/ED-psychopathology remission rates at 4-week follow-up, applying German-AN-S3-guidelines-based vs. DSM-5-based remission criteria.

As main study findings we observed body weight metrics increase and ED-psychopathology reduction from admission to IMT (lasting on average 3.9 months) to 4-week follow-up, with greater effect sizes for body weight metrics compared to ED-psychopathology improvement. Body weight metrics and ED-psychopathology severity were not associated, neither cross-sectionally at baseline nor at 4-week follow-up, nor regarding change values over time. A greater increase in BMI *z*-score between baseline and 4-week follow-up was independently predicted by lower baseline BMI *z*-score and less baseline psychiatric comorbidities, explaining 54.5% of the variance in change in BMI *z*-score. Greater reduction in EDE-Q *global score* between baseline and 4-week follow-up was independently predicted by typical AN vs. atypical AN diagnosis, higher baseline BMI *z*-score, and higher EDE-Q *restraint* subscore, explaining 37.6% of the variance in change in EDE-Q *global score*. Full and partial remission rates varied substantially depending on applied German-AN-S3-guideline-based vs. DSM-5-based remission criteria.

Although 4-week follow-up body weight metrics were still below the German-AN-S3-guidelines-recommended ≥ 25th BMI percentile in 77.5% patients (achieving the DSM-5-required ≥ 5th BMI percentile in 80.0% of patients), the highest effect sizes were found for body weight metric increase from baseline to 4-week follow-up, consistent with prior ED inpatient treatment studies [9,50,51,52,53,54,55,56]. Baseline EDE-Q subscores *restraint* and *eating concern* were lower than typically reported for patients with restricting EDs [27]—potentially due to baseline assessment execution in an inpatient psychiatric multimodal treatment setting preventing dietary restraint—yet were still increased compared to community norms [27]. The 4-week follow-up EDE-Q *global score* was ≤1*SD* of community norms [27] in 65.0% (26/40) of patients, with a smaller, but still large effect size compared to body weight metrics improvement. Although 4-week follow-up EDE-Q subscores ranged within ≤1*SD* of community norms, effect sizes were lower for subscores *restraint* and *eating concern* compared to *weight*/*shape concern* decrease.

While ED treatments target both body weight and ED-psychopathology, only body weight outcomes have been systematically reported in inpatient treatment studies [57], while data on outcomes in ED-psychopathology are scarcer. Nevertheless, our findings contrast with results by Fennig et al. [9], and Gowers et al. [13] who found little to no improvement in ED cognitions and perceptions in adolescents with AN after inpatient treatment. However, our results confirm prior data showing decreased ED-psychopathology after inpatient treatment in adolescents with mixed EDs [58,59], and improved body distortion in patients with AN [14,15]. Implementing therapeutic techniques focusing on core cognitions could lead to ED-psychopathology improvement [9,60,61]. In our study, weight gain/week during IMT was inversely associated with 4-week follow-up ED-psychopathology. Evidence across ED diagnoses [22,62,63,64,65] supports the prognostic importance of early and rapid weight gain regarding ED-psychopathology improvement and full remission, although interdependences and the sequencing of faster weight gain and ED-psychopathology decrease require further evaluation.

The lack of cross-sectional significant correlations between body weight metrics and ED-psychopathology in our study sample is suggestive but not definitive and needs to be replicated in larger and independent samples. Yet the lack of associations contrasts with the general assumption of lower body weight being associated with, or a result of, higher ED-psychopathology. Similar to our findings, previous studies found ED-psychopathology in patients with AN dissociable from DSM-5 BMI-based severity specifiers [66,67]. Quadflieg et al. [68] found higher baseline ED-psychopathology in adolescents with AN reaching good vs. poor body weight outcome at discharge from inpatient treatment. Garber et al. [69] reported neither body weight at discharge nor weight gain being associated with EDE-Q scores at discharge from inpatient treatment in adults with AAN; yet in adults with AN, discharge body weight and weight gain were associated with EDE-Q *restraint* at discharge. These conflicting results seem to indicate, especially in an IMT environment where medical professionals jointly target body weight restoration more than ED-psychopathology, body weight changes may not correlate well with ED-psychopathology severity, yet differences between adults and adolescents with AN may also play a role. Complex body weight–ED-psychopathology relationships seem to be affected by various (neuro)biological factors, including neurobiological vulnerability [70,71], endocrine secretory dynamics [72,73,74,75], premorbid body composition [76,77], and weight suppression (difference between highest and current body weight metrics) [78,79,80,81]. Body fat rather than body weight might be linked with ED-psychopathology via reductions in leptin secretion in patients with AN [82]. Furthermore, behavioral/psychological factors (e.g., amount/speed of weight loss; body image disturbance) [83,84,85], structural determinants (e.g., illness duration; functional impairment [86], and life events [87]) need to be considered.

Different predictors for outcomes in treatment of AN have been previously established, with mixed results [54,59,65,68,88,89,90,91,92,93,94,95]. A meta-analysis identified several positive outcome predictors for the treatment of patients with AN, such as higher body weight metrics, lower ED-psychopathology, fewer psychiatric comorbidities, and higher motivation to change, with greater symptom change early during treatment as a mediator of better outcomes [88]. A review of predictors of treatment outcomes in patients with mixed EDs found that lower BMI had a negative impact on treatment outcome, but found mixed results for the impact of baseline ED symptom severity and psychiatric comorbidities [93]. In a sample of 653 adolescents with AN and AAN receiving outpatient family-based treatment [94], lower baseline BMI, higher ED severity, and psychiatric comorbidities conditions predicted longer duration to treatment completion, with BMI four-week intra-treatment being a key predictor [94]. Consistent with the finding in a sample of 4863 patients with AN (age 22.3 ± 9.8 years; 97% female) receiving inpatient treatment [95] and corresponding with more room for weight gain until reaching a target body weight required for discharge, in our sample, lower baseline BMI *z*-score predicted greater ultimate change in BMI *z*-score between baseline and 4-week follow-up. Across ED diagnoses, in a large sample of 1971 adolescents receiving higher levels of care, psychiatric comorbidities predicted less weight gain [59]. Similar to prior results, in our study, fewer psychiatric comorbidities at baseline predicted a greater increase in BMI *z*-score.

When comparing illness course and outcome between patients with an AN vs. AAN diagnosis, previous data are limited and have rarely specifically reported on changes in ED-psychopathology [96,97]. In our sample, typical AN-R/-BP vs. AAN diagnosis was an independent predictor for greater improvement in ED-psychopathology. Inconsistent with this finding, a previous study in adolescents and adults with AN (*n* = 319) and AAN (*n* = 67) treated in a partial hospitalization program found faster ED-psychopathology reduction in patients with AAN from baseline to discharge, and no group differences in ED-psychopathology at discharge between AN vs. AAN diagnosis [98]. However, these findings need further confirmation in larger study samples assessing the course, outcome, and treatment response of patients with AAN beyond the consideration of only baseline characteristics [99]. In our study sample, higher baseline EDE-Q subscore *restraint* was associated with a greater improvement in ED-psychopathology between baseline and 4-week follow-up, but not with greater increase in BMI *z*-score. This result confirms the prior data [54] of 238 adolescents with AN receiving inpatient treatment, in that BMI gain did not significantly differ between patients with vs. without a clinically significant reduction in ED-psychopathology. In prior studies, the duration of IMT, comorbid depression, and body dissatisfaction were significantly associated with less improvements in ED-psychopathology [54]. This finding was not replicated in our small study sample with more broadly assessed baseline and treatment variables. Similar to our finding, however, a study of 1971 adolescents with mixed EDs (AN-R, AN-BP, bulimia nervosa, or other specified feeding or eating disorder) reported that receiving higher levels of care, and having higher admission EDE-Q *global scores* were each associated with greater improvements in cognitive ED symptoms [59]. Another study in 121 adolescents with AN-R/-BP found that higher baseline severity of ED symptoms predicted the improvement of ED-psychopathology [100]. While greater improvements in ED-psychopathology might partly be an effect of regression to the mean, it nevertheless could indicate that patients with higher symptom severity, e.g., higher EDE-Q *restraint*, improve more during IMT which specifically focuses both on the improvement of body weight and ED-psychopathology.

In comparing these results of prior studies with our study result, it is important to note that previous studies focused on predictors for absolute outcomes at endpoint and not on changes in body weight metrics or ED-psychopathology, although a focus on change might help to detect the predictors of treatment response more precisely, given that baseline differences would be accounted for.

In our study, applying two different criteria systems, full remission rates at 4-week follow-up varied considerably. According to DSM-5 criteria, half of the patients achieved full remission compared to only one out of 8 according to German-AN-S3-guidelines-based criteria, but the threshold for the absence of underweight was low, with ≥5th BMI percentile as per DSM-5 compared to requiring ≥25th BMI percentile as per German-AN-S3 guidelines. Partial body weight remission varied between 10.0% applying a stricter German-AN-S3-guideline and 30.0% as per DSM-5 body weight remission criterion. Although we utilized the same ED-psychopathology remission criterion, more patients (52.5% vs. 15.0%) were partially ED-psychopathology remitted according to German-AN-S3-guidelines-based vs. DSM-5 criteria, simply because less patients reached full remission according to German-AN-S3-guidelines. Finally, after almost four months of IMT and 4-week follow-up, only 5.0% of patients according to DSM-5, and still 25.0% according to German-AN-S3-guidelines, remained in non-remission at 4-week follow-up. Due to the rarity of inpatient treatment studies in adolescents with restricting EDs reporting on body weight- and ED-psychopathology-related remission criteria [25,57,101], and even fewer studies reporting German-AN-S3-guidelines-based criteria, DSM-5 remission criteria, or comparing both, the comparability of our findings is limited. Applying DSM-5 remission criteria but setting the healthy body weight at ≥10th BMI percentile [21], Mairhofer et al. [25], found remission in 23.2% of 126 adolescents with AN at the end of IMT. Solmi et al. [5] found in their recent comprehensive systematic review an overall recovery rate of only 45.0% in patients with AN (independent of lifetime hospitalization status) based on Morgan–Russell criteria [102], but at a long follow-up (72.8 ± 76.8 months), while overall meta-analytic chronicity rate in patients with AN was 23.0% at 80.4 ± 79.1 months follow-up. Mekori et al. [90] categorized 31.8% of female adolescents with EDs as remitted one year after inpatient treatment, defining ≥85% EBW as body weight remission, and the absence of ED-related behaviors as ED-psychopathology remission. Le Grange et al. [29] found remission rates varying between 21.7 and 87.7% in adolescents with AN after outpatient FBT, depending on 11 different body weight and/or ED-psychopathology related definitions of remission. Similarly, applying seven different remission criteria, Couturier et al. [16] found full remission in 3–96%. Consistent with prior studies, divergent full and partial remission rates in our study highlight the need for uniformly defined and applied criteria sets pertaining to body weight, ED-related cognitions and behaviors, to allow a meaningful comparison of results. This issue has been raised before [5,16,29,30]. The interesting disconnect in our sample between 80.0% in BMI percentile remission when using ≥5th BMI percentile threshold—which corresponds to a very lenient definition of being within the 95% or 1.68 *SD* of normal body weight, and 65.0% of patients being in ED-psychopathology remission when being within 1 *SD* (or 68th percentile) of EDE-Q *global score*, which signifies a less lenient threshold—indicates the need to calibrate and harmonize cut-offs across different outcome domains when defining remission criteria for each domain.

**Strengths and limitations**: To the best of our knowledge, no prior study systematically compared body weight and ED-psychopathology changes during IMT and after subsequent brief ambulatory care in adolescents with AN and AAN. Some study limitations need to be considered. First, the main limitation of this study is the small sample size. Therefore, the lack of significant associations between body weight and ED-psychopathology needs to be interpreted cautiously, given the low number of patients in this analysis and the related reduced power for such analyses, which is why our findings need to be replicated in larger and independent samples. A post hoc power analysis yielded that with 40 patients, α = 0.05, and β = 0.80, we were able to detect a minimum correlation coefficient of 0.43, which is in the middle of a moderate effect size between 0.30 and 0.60 [49]. Second, there was no control group. However, the main study focus on relationships between body weight metrics and ED-psychopathology changes does not require a control group as this relationship does not apply to healthy controls. Third, lacking information on the patients who declined study participation meant we were unable to report on selection bias. Study dropouts had a shorter IMT duration, which might indicate the reduced generalizability of the analyzed completer sample, as premature discharge from IMT against medical advice could be associated with potentially negative consequences for course of illness, which would have been undetected due to study dropout. However, attrition bias for study completers was considered low (attrition rate of 9%). Fourth, the inclusion of AAN could make the results less readily applicable to studies with AN-R and AN-BP patients only, and the sample size was too small for meaningful subanalyses of (A)AN subsamples. Fifth, we only studied adolescents requiring IMT as per German-AN-S3-criteria. Therefore, we cannot comment on body weight metrics and ED-psychopathology changes and relationships in adolescents treated in outpatient settings or different healthcare systems, or in adults. Sixth, combining various psychological interventions and prescriptive nutritional strategies applied during IMT complicates replication. Yet, it depicts a clinical “real world” standard of care in a German psychiatric inpatient setting, which should enhance the generalizability of the results. Seventh, due to lacking EDE-Q data at discharge, remission and body weight/ED-psychopathology interaction analyses could not be conducted at EOT, but only after four weeks of uncontrolled and unspecified ambulatory care, leaving a gap in understanding change trajectory patterns. This limitation constricts conclusions regarding immediate treatment effects. Including end of treatment assessments in future studies would allow for a more precise characterization of patterns of change and treatment response over time. Yet four weeks is a short duration, so results can still be considered as effects of IMT on ED-pathology. Finally, this study included a relatively short follow-up assessment conducted four weeks post-discharge from inpatient psychiatric multimodal treatment. This follow-up period may be considered particularly brief in the context of ED-psychopathology, which typically changes more slowly than weight-related outcomes. This limitation has important implications for the interpretation of our findings, as longer-term ambulatory follow-up data on body weight metrics and ED-psychopathology changes, interactions, and remission patterns would be highly valuable. However, the primary aim of the present study was to investigate early post-discharge effects, while longer-term follow-up data in a smaller subset will be focused on separately.

## 5. Conclusions

The main present-study findings contradict prior studies stating that inpatient treatment has no impact on ED cognitions and perceptions in adolescents with AN [9], or claiming that ED cognition and perception remission does not occur until an average of 23–24 months after the beginning of treatment [10]. Our findings suggest that IMT, aiming not only at body weight restoration, but also providing targeted therapeutic interventions to address ED-psychopathology, can lead not only to short-term weight gain, but has also positive effects on self-reported ED-related behavior, cognitions, and perceptions. In accordance with previous findings [22,63,65,66], greater weight gain per week had a positive impact on ED-psychopathology. However, complex interactions between body weight and ED-psychopathology severity and change during inpatient and subsequent outpatient care require further investigation in order to identify treatment targets to improve short- and long-term outcomes for this patient population. In particular, further research is needed on (i) how to best measure ED-psychopathology while taking into consideration the role of anosognosia vs. illness-insight, and (ii) how to parse average scores and correlations into subgroups given the heterogeneity of the severity of each ED component and of the weightED-psychopathology trajectories. Potentially relevant trajectory subgroups include (i) improvement in ED-psychopathology precedes and drives weight gain, (ii) weight gain is not paralleled by the same degree of ED-psychopathology improvement, or (iii) weight gain worsens ED-psychopathology. Additionally, research is needed on how to best achieve the remission of body weight and ED-psychopathology since mere refeeding does not seem to sufficiently drive improvement in ED-psychopathology. Finally, there is the need for an international expert consensus on the operational definition of remission in patients with AN and AAN.

## Figures and Tables

**Table 1:** Baseline, clinical, and treatment characteristics of total sample and study completers.

	Total Sample (*n* = 56, 100%)	Study Completers (*n* = 40, 71.4%)
Sex (*n*, %)		
Female	51 (91.1)	36 (90.0)
Male	5 (8.9)	4 (10.0)
Age, years, mean ± *SD* (range)	15.4 ± 1.6 (12.0–18.3)	15.6 ± 1.5 (12.1–17.8)
Duration of illness, months, median [IQR]	11.5 [7.0–18.8]	12.0 [7.3–18.8]
AN subtype (*n*, %)		
AN-R	29 (51.8)	20 (50.0)
Atypical AN	19 (33.9)	14 (35.0)
AN-BP	8 (14.3)	6 (15.0)
Menstrual status (*n*, %)		
Secondary amenorrhea	39 (69.6)	26 (65.0)
Primary amenorrhea	6 (10.7)	4 (10.0)
Male sex	5 (8.9)	4 (10.0)
Contraception	4 (7.1)	4 (10.0)
Regular menses	2 (3.6)	2 (5.0)
Comorbid psychiatric diagnosis (*n*, %)		
Depression/dysthymia	22 (39.3)	15 (37.5)
Personality disorder/trait	6 (10.7)	3 (7.5)
Obsessive–compulsive disorder	5 (8.9)	3 (7.5)
Anxiety disorder	3 (5.4)	3 (7.5)
Psychotropic medication (*n*, %)		
Antidepressants	6 (10.7)	3 (7.5)
Antipsychotics	2 (3.6)	1 (2.5)
Premorbid body weight, kg, median [IQR]	54 [48–63]	54 [48–63]
Baseline body weight metrics		
BMI percentile, median [IQR]	1 [<1–5]	<1 [<1–3]
BMI *z*-score, mean ± *SD* (range)	−2.42 ± 1.08 (−4.46–−0.04)	−2.59 ± 1.07 (−4.46–−0.04)
%mBMI, median [IQR]	75.4 [70.9–82.7]	74.3 [69.7–80.6]
Baseline EDE-Q scores		
*Global score*, median [IQR]	3.67 [2.22–5.00], *n* = 50 ^a^	3.26 [1.84–4.94], *n* = 37 ^a^
*Restraint*, median [IQR]	3.10 [1.20–3.10], *n* = 50 ^a^	2.80 [1.00–4.68], *n* = 37 ^a^
*Eating concern*, mean ± *SD* (range)	2.76 ± 1.55 (0–5.60), *n* = 44 ^a,b^	2.63 ± 1.60 (0–5.60), *n* = 35 ^a,b^
*Weight concern*, median [IQR]	3.80 [2.15–5.20], *n* = 50 ^a^	3.60 [2.10–5.10], *n* = 37 ^a^
*Shape concern*, median [IQR]	4.31 [2.63–5.75], *n* = 50 ^a^	4.25 [2.63–5.56], *n* = 37 ^a^
IMT duration, days, median [IQR]	108.0 [78.3–140.3]	117.5 [90.0–150.3]

AN, anorexia nervosa. AN-R, anorexia nervosa, restricting subtype. AN-BP, anorexia nervosa, binge–purge subtype. BMI, body mass index. EDE-Q, Eating Disorder Examination Questionnaire. IMT, inpatient psychiatric multimodal treatment. IQR, interquartile range. kg, kilogram. *n*, number. *p*, probability value (significance tests: *t*-test for independent samples, Fisher Exact Test or contingency coefficient; choice depending on data level). *SD*, standard deviation. ^a^ missing *n* due to missing EDE-Q data. ^b^ missing *n* due to missing 2nd page of EDE-Q. %mBMI, percent median body mass index.

**Table 2:** Body weight metrics and eating disorder psychopathology at baseline and four weeks post-discharge from inpatient psychiatric multimodal treatment.

	Baseline (*n* = 40)	4-Week Follow-Up (*n* = 40)	*p*-Value	Effect Size
Body weight metrics, median [IQR]				
BMI percentile	<1 [<1–3]	16 [7–22]	<0.001 **	*r* = 0.82
BMI *z*-score	−2.61 [−3.29–−1.91]	−1.04 [−1.49–−0.78]	<0.001 **	*r* = 0.85
%mBMI	74.3 [69.7–80.6]	88.1 [84.0–92.1]	<0.001 **	*r* = 0.86
EDE-Q scores, median [IQR]				
*Global score*	3.26 [1.84–4.94], *n* = 37 ^a^	1.81 [1.36–3.52], *n* = 40	<0.001 **	*r* = 0.66
*Restraint*	2.80 [1.00–4.66], *n* = 37 ^a^	1.60 [0.70–2.95], *n* = 40	0.004 **	*r* = 0.47
*Eating concern*	2.60 [1.20–3.80], *n* = 35 ^a,b^	1.80 [0.90–2.70], *n* = 37 ^b^	0.005 **	*r* = 0.50
*Weight concern*	3.60 [2.10–5.10], *n* = 37 ^a^	1.80 [1.20–4.00], *n* = 40	<0.001 **	*r* = 0.60
*Shape concern*	4.25 [2.63–5.56], *n* = 37 ^a^	2.44 [1.50–4.97], *n* = 40	<0.001 **	*r* = 0.68

Abbreviations: 4-week follow-up, four weeks post-discharge from inpatient psychiatric multimodal treatment; %mBMI, percent median body mass index; BMI, body mass index; EDE-Q, Eating Disorder Examination Questionnaire; N, number; *p*, probability value (significance tests: *t*-Test for paired samples/Wilcoxon Test depending on data level); ^a^ missing *n* due to missing EDE-Q data; ^b^ missing *n* due to missing 2nd page of EDE-Q; ** *p* < 0.01. r, effect size (<0.3 small effect size, 0.3–0.6 medium effect size, >0.6 large effect size).

**Table 3:** Association between body weight metrics at baseline and four weeks post-discharge from inpatient psychiatric multimodal treatment with eating disorder psychopathology at baseline and four weeks post-discharge from inpatient psychiatric multimodal treatment.

	Baseline Body Weight Metrics (*n* = 40)			4-Week Follow-Up Body Weight Metrics (*n* = 40)		
	%mBMI	BMI Percentile	BMI *z*-Score	%mBMI	BMI Percentile	BMI *z*-Score
	median [IQR]	median [IQR]	mean ± *SD* (range)	mean ± *SD* (range)	median [IQR]	median [IQR]
	74.3 [69.7–80.6]	<1 [<1–3]	−2.59 ± 1.07 (−4.46–−0.04)	88.1 ± 8.1 (71.4–110.6)	16 [7–22]	−1.04 [−1.49–−0.78]
Baseline EDE-Q scores						
*Global score*, mean ± *SD* (range) 3.26 ± 1.61 (0.39–5.72), *n* = 37 ^a^	*r_sp_* = 0.194 *p* = 0.250	*r_sp_* = 0.065 *p* = 0.701	*r* = 0.254 *p* = 0.129	*r* = 0.229 *p* = 0.172	*r_sp_* = 0.114 *p* = 0.503	*r_sp_* = 0.093 *p* = 0.583
*Restraint*, median [IQR] 2.80 [1.00–4.68], *n* = 37 ^a^	*r_sp_* = 0.089 *p* = 0.599	*r_sp_* = −0.058 *p* = 0.735	*r* = 0.114 *p* = 0.501	*r* = 0.199 *p* = 0.238	*r_sp_* = 0.176 *p* = 0.297	*r_sp_* = 0.148 *p* = 0.381
*Eating concern*, mean ± *SD* (range) 2.63 ± 1.60 (0–5.60), *n* = 35 ^a,b^	*r_sp_* = 0.106 *p* = 0.544	*r_sp_* = −0.034 *p* = 0.845	*r* = 0.145 *p* = 0.405	*r* = 0.178 *p* = 0.306	*r_sp_* = 0.078 *p* = 0.657	*r_sp_* = 0.040 *p* = 0.819
*Weight concern*, mean ± *SD* (range) 3.45 ± 1.83 (0–6.00), *n* = 37 ^a^	*r_sp_* = 0.241 *p* = 0.150	*r_sp_* = 0.132 *p* = 0.437	*r* = 0.287 *p* = 0.085	*r* = 0.162 *p* = 0.338	*r_sp_* = 0.069 *p* = 0.686	*r_sp_* = 0.046 *p* = 0.787
*Shape concern*, median [IQR] 4.25 [2.63–5.56], *n* = 37 ^a^	*r_sp_* = 0.18 *p* = 0.277	*r_sp_* = 0.120 *p* = 0.481	*r* = 0.192 *p* = 0.254	*r* = 0.127 *p* = 0.453	*r_sp_* = 0.113 *p* = 0.506	*r_sp_* = 0.086 *p* = 0.613
4-week follow-up EDE-Q scores						
*Global score*, median [IQR] 1.81 [1.36–3.52], *n* = 40	*r_sp_* = 0.010 *p* = 0.950	*r_sp_* = −0.085 *p* = 0.602	*r* = 0.018 *p* = 0.912	*r* = −0.025 *p* = 0.880	*r_sp_* = −0.042 *p* = 0.796	*r_sp_* = −0.046 *p* = 0.780
*Restraint*, median [IQR] 1.60 [0.70–2.95], *n* = 40	*r_sp_* = 0.074 *p* = 0.648	*r_sp_* = −0.047 *p* = 0.774	*r* = 0.093 *p* = 0.568	*r* = −0.024 *p* = 0.881	*r_sp_* = −0.049 *p* = 0.763	*r_sp_* = −0.056 *p* = 0.733
*Eating concern*, median [IQR] 1.80 [0.90–2.70], *n* = 37 ^b^	*r_sp_* = −0.061 *p* = 0.722	*r_sp_* = −0.080 *p* = 0.639	*r* = −0.048 *p* = 0.778	*r* = 0.115 *p* = 0.499	*r_sp_* = 0.057 *p* = 0.738	*r_sp_* = 0.074 *p* = 0.664
*Weight concern*, median [IQR] 1.80 [1.20–4.00], *n* = 40	*r_sp_* = 0.064 *p* = 0.695	*r_sp_* = −0.025 *p* = 0.881	*r* = 0.071 *p* = 0.663	*r* = 0.026 *p* = 0.875	*r_sp_* = 0.008 *p* = 0.963	*r_sp_* = 0.010 *p* = 0.951
*Shape concern*, median [IQR] 2.44 [1.50–4.97], *n* = 40	*r_sp_* = 0.095 *p* = 0.560	*r_sp_* = 0.015 *p* = 0.926	*r* = 0.098 *p* = 0.548	*r* = 0.059 *p* = 0.719	*r_sp_* = 0.076 *p* = 0.643	*r_sp_* = 0.053 *p* = 0.744

Abbreviations: 4-week follow-up, four weeks post-discharge from inpatient psychiatric multimodal treatment; BMI, body mass index; %mBMI, percent median body mass index; EDE-Q, Eating Disorder Examination Questionnaire; *p*, probability value; *r_sp_*, Spearman’s rho. ^a^ missing *n* due to missing EDE-Q data. ^b^ missing *n* due to missing 2nd page of EDE-Q.

**Table 4:** Correlations between baseline and clinical characteristics of the study sample, change in body mass index *z*-score, and change in Eating Disorder Examination Questionnaire *global score* from baseline to four weeks post-discharge from inpatient psychiatric multimodal treatment.

	Change in BMI *z*-Score from Baseline to 4-Week Follow-Up (*n* = 40)	*p*-Value	Change in EDE-Q *Global Score* from Baseline to 4-Week Follow-Up (*n* = 37 ^a^)	*p*-Value
Sex	−0.061	0.711	−0.090	0.597
Admission age (years)	0.062	0.702	0.058	0.734
Age of AN onset (years)	0.177	0.274	0.135	0.427
Number of previous inpatient treatments	0.135	0.405	−0.004	0.981
Duration of illness (months)	−0.049	0.764	−0.052	0.759
Typical AN-R/AN-BP vs. AAN	−0.314	0.049 *	−0.387	0.018 *
Comorbid psychiatric diagnosis				
Depression/dysthymia	−0.353	0.025 *	0.180	0.287
Personality disorder/trait	0.032	0.842	−0.001	0.996
Obsessive–compulsive disorder	−0.025	0.878	0.062	0.717
Anxiety disorder	−0.197	0.224	0.303	0.068
Number of psychiatric comorbidities	−0.333	0.036	0.281	0.092
Psychotropic medications				
Antidepressants (yes or no)	−0.058	0.724	0.201	0.233
Antipsychotics (yes or no)	0.221	0.171	−0.078	0.645
Baseline body weight metrics				
BMI percentile	−0.504 (Kendall’s Tau)	<0.001 **	0.128	0.308
BMI *z*-score	−0.715	<0.001 **	0.279	0.094
%mBMI	−0.689	<0.001 **	0.259	0.121
Baseline EDE-Q scores				
*Global score*	−0.146	0.389	0.520	<0.001 **
*Restraint*	0.004	0.979	0.480	0.003 **
*Eating concern*	−0.043	0.807	0.546	<0.001 ** (*n* = 35 ^a,b^)
*Weight concern*	−0.254	0.130	0.464	0.004 **
*Shape concern*	−0.201	0.232	0.499	0.002 **
IMT duration (days)	0.201	0.213	−0.217	0.197

Abbreviations: AN, anorexia nervosa; AAN, atypical anorexia nervosa; AN-R, anorexia nervosa, restricting subtype; AN-BP, anorexia nervosa, binge–purge subtype; BMI, body mass index; EDE-Q, Eating Disorder Examination Questionnaire; IMT, inpatient psychiatric multimodal treatment; N, number; *p*, probability value; ^a^ missing *n* due to missing EDE-Q data; ^b^ missing *n* due to missing 2nd page of EDE-Q; %mBMI, percent median body mass index. Values are frequencies (percent) or means ± standard deviation (*SD*) (range) in case of normal distribution or median [inter quartile range] (range) for not normally distributed variables. * *p* < 0.05; ** *p* < 0.01.

**Table 5:** Final multivariable stepwise backward elimination regression model of correlates independently associated with BMI *z*-score increase from baseline to four weeks post-discharge from inpatient psychiatric multimodal treatment.

Characteristic	Unstandardized Coefficients		Standardized Coefficients	T	Sig.	95% Confidence Interval for B		Collinearity Statistics	
	B	Std. Error	β			Lower Bound	Upper Bound	Tolerance	VIF
(Constant)	0.139	0.277		0.501	0.619	−0.422	0.699		
Baseline BMI *z*-score	−0.566	0.090	−0.683	−6.262	<0.001	−0.749	−0.383	0.982	1.019
Number of psychiatric comorbidities	−0.317	0.144	−0.240	−2.204	0.034	−0.608	−0.026	0.982	1.019

Dependent variable: change in BMI *z*-score. BMI, body mass index. β, regression coefficient. Std. Error, standard error. Sig., significance. VIF, variance inflation factor. Excluded variables during the backward elimination selection process: baseline comorbid psychiatric diagnosis of depression or dysthymia, typical anorexia nervosa vs. atypical anorexia nervosa diagnosis.

**Table 6:** Final multivariable regression model of independent correlates associated with reduction (i.e., improvement) in Eating Disorder Examination Questionnaire *global score* from baseline to four weeks post-discharge from inpatient psychiatric multimodal treatment.

Characteristic	Unstandardized Coefficients		Standardized Coefficients	T	Sig.	95% Confidence Interval for B		Collinearity Statistics	
	B	Std. Error	β			Lower Bound	Upper Bound	Tolerance	VIF
(Constant)	2.185	0.735		2.973	0.005	0.690	3.681		
Typical AN vs. AAN	−1.224	0.404	−0.457	−3.027	0.005	−2.046	−0.401	0.759	1.317
Higher baseline BMI *z*-score	0.508	0.191	0.395	2.663	0.012	0.120	0.896	0.787	1.217
Higher baseline EDE-Q *restraint*	0.199	0.097	0.293	2.053	0.048	0.002	0.397	0.850	1.177

Dependent Variable: change in EDE-Q *global score*. Abbreviations: AN, anorexia nervosa; AN-R, anorexia nervosa restricting subtype; AN-BP, anorexia nervosa binge–purge subtype; AAN, atypical anorexia nervosa; EDE-Q, Eating Disorder Examination Questionnaire; B, regression coefficient; β, regression coefficient; Std. Error, standard error; Sig., significance; VIF, variance inflation factor. Excluded variables during the backward elimination selection process: baseline EDE-Q global score, subscores EDE-Q *eating concern*, EDE-Q *weight concern*, and EDE-Q *shape concern*, baseline comorbid anxiety disorder, baseline number of psychiatric comorbidities.

## Data Availability

Data supporting the study findings are available from the corresponding author upon reasonable request.

## Abbreviations

The following abbreviations are used in this manuscript:

USA	United States of America
ED	Eating disorder
AN	Anorexia nervosa
4-week follow-up	Four weeks post-discharge from inpatient psychiatric multimodal treatment
IMT	Inpatient psychiatric multimodal treatment
BMI	Body mass index
AN-R	Anorexia nervosa-restricting type
AN-BP	Anorexia nervosa-binge–purge type
AAN	Atypical anorexia nervosa
EDE-Q	Eating Disorder Examination Questionnaire, German Version
IQR	Interquartile range
*SD*	Standard deviation
EBW	Expected body weight
EOT	End of inpatient psychiatric multimodal treatment
kg	Kilogram

## Appendix A

**Table A1:** Baseline, clinical and treatment characteristics of the study sample.

	Total Sample (*n* = 56, 100%)	Study Completers (*n* = 40, 71.4%)	Study Dropouts (*n* = 16, 28.6%)	*p*-Value: Study Completers vs. Study Dropouts
Sex (*n*, %)				1.000
Female	51 (91.1)	36 (90.0)	15 (93.8)	
Male	5 (8.9)	4 (10.0)	1 (6.3)	
Age, years, mean ± *SD* (range)	15.4 ± 1.6 (12.0–18.3)	15.6 ± 1.5 (12.1–17.8)	14.9 ± 1.7 (12.0–18.3)	0.104
Duration of illness, months, median [IQR]	11.5 [7.0–18.8]	12.0 [7.3–18.8]	9.0 [7.0–22.5]	0.820
AN subtype (*n*, %)				1.000
AN-R	29 (51.8)	20 (50.0)	9 (56.3)	
Atypical AN	19 (33.9)	14 (35.0)	5 (31.3)	
AN-BP	8 (14.3)	6 (15.0)	2 (12.5)	
Menstrual status (*n*, %)				0.766
Secondary amenorrhea	39 (69.6)	26 (65.0)	13 (81.3)	
Primary amenorrhea	6 (10.7)	4 (10.0)	2 (12.5)	
Male sex	5 (8.9)	4 (10.0)	1 (6.3)	
Contraception	4 (7.1)	4 (10.0)	0	
Regular menses	2 (3.6)	2 (5.0)	0	
Comorbid psychiatric diagnosis (*n*, %)				0.245
Depression/dysthymia	22 (39.3)	15 (37.5)	7 (43.8)	0.765
Personality disorder/trait	6 (10.7)	3 (7.5)	3 (18.8)	0.338
Obsessive–compulsive disorder	5 (8.9)	3 (7.5)	2 (12.5)	0.617
Anxiety disorder	3 (5.4)	3 (7.5)	0	0.550
Psychotropic medications (*n*, %)				
Antidepressants	6 (10.7)	3 (7.5)	2 (12.5)	0.338
Antipsychotics	2 (3.6)	1 (2.5)	1 (6.3)	0.494
Premorbid body weight, kg, median [IQR]	54 [48–63]	54 [48–63]	55 [48–64]	0.838
Baseline body weight metrics				
BMI percentile, median [IQR]	1 [<1–5]	<1 [<1–3]	3 [<1–13]	0.078
BMI *z*-score, mean ± *SD* (range)	−2.42 ± 1.08 (−4.46–−0.04)	−2.59 ± 1.07 (−4.46–−0.04)	−1.99 ± 1.02 (−3.81–−0.27)	0.060
%mBMI, median [IQR]	75.4 [70.9–82.7]	74.3 [69.7–80.6]	80.1 [73.8–80.1]	0.048 *
Baseline EDE-Q scores				
*Global score*, median [IQR]	3.67 [2.22–5.00], *n* = 50 ^a^	3.26 [1.84–4.94], *n* = 37 ^a^	4.00 [2.65–5.22], *n* = 13 ^a^	0.213
*Restraint*, median [IQR]	3.10 [1.20–3.10], *n* = 50 ^a^	2.80 [1.00–4.68], *n* = 37 ^a^	4.00 [1.60–5.30], *n* = 13 ^a^	0.273
*Eating concern*, mean ± *SD* (range)	2.76 ± 1.55 (0–5.60), *n* = 44 ^a,b^	2.63 ± 1.60 (0–5.60), *n* = 35 ^a,b^	3.24 ± 1.31 (0.80–4.60), *n* = 9 ^a,b^	0.298
*Weight concern*, median [IQR]	3.80 [2.15–5.20], *n* = 50 ^a^	3.60 [2.10–5.10], *n* = 37 ^a^	4.40 [2.10–4.40], *n* = 13 ^a^	0.445
*Shape concern*, median [IQR]	4.31 [2.63–5.75], *n* = 50 ^a^	4.25 [2.63–5.56], *n* = 37 ^a^	5.63 [2.63–5.94], *n* = 13 ^a^	0.184
IMT duration, days, median [IQR]	108.0 [78.3–140.3]	117.5 [90.0–150.3]	78.5 [48.0–107.5]	0.004 **

Abbreviations: AN, anorexia nervosa. AN-R, anorexia nervosa, restricting subtype. AN-BP, anorexia nervosa, binge–purge subtype. BMI, body mass index. EDE-Q, Eating Disorder Examination Questionnaire. IMT, inpatient psychiatric multimodal treatment. IQR, interquartile range. kg, kilogram. N, number. *p*, probability value (significance tests: *t*-test for independent samples, Fisher Exact Test or contingency coefficient; choice depending on data level). *SD*, standard deviation. ^a^ missing n due to missing EDE-Q data. ^b^ missing n due to missing 2nd page of EDE-Q. %mBMI, percent median body mass index. * *p* < 0.05. ** *p* < 0.01.
